# Supplementary material for: Modeling glioblastoma heterogeneity as a dynamic network of cell states
Source: Mol Syst Biol. 2021 Sep 16;17(9):e10105. doi: 10.15252/msb.202010105 (PMC8444284; doi:10.15252/msb.202010105)
Supplement: Supplementary file 6 — Source Data for Figure 5 [file MSB-17-e10105-s004.zip › Figure5A_sourcedata/GSEA_3017/nosigdb_stateD.GseaPreranked.1622795010397/SEURAT_CELLCYCLE.html]

Details for gene set SEURAT\_CELLCYCLE[GSEA]

|  || Dataset | state23017 |
| Phenotype | NoPhenotypeAvailable |
| Upregulated in class | na\_neg |
| GeneSet | SEURAT\_CELLCYCLE |
| Enrichment Score (ES) | -0.553409 |
| Normalized Enrichment Score (NES) | -2.8001726 |
| Nominal p-value | 0.0 |
| FDR q-value | 0.0 |
| FWER p-Value | 0.0 |
Table: GSEA Results Summary

  

Fig 1: Enrichment plot: SEURAT\_CELLCYCLE      
 Profile of the Running ES Score & Positions of GeneSet Members on the Rank Ordered List

  

| PROBE | GENE SYMBOL | GENE\_TITLE | RANK IN GENE LIST | RANK METRIC SCORE | RUNNING ES | CORE ENRICHMENT || 1 | BIRC5 |  |  | 130 | 0.271 | -0.0964 | No |
| 2 | MKI67 |  |  | 206 | -0.254 | -0.1494 | No |
| 3 | MCM2 |  |  | 226 | -0.256 | -0.1576 | No |
| 4 | HMMR |  |  | 319 | -0.266 | -0.2238 | No |
| 5 | UHRF1 |  |  | 438 | -0.278 | -0.3105 | No |
| 6 | ECT2 |  |  | 481 | -0.283 | -0.3363 | No |
| 7 | WDR76 |  |  | 557 | -0.292 | -0.3883 | No |
| 8 | SLBP |  |  | 573 | -0.295 | -0.3922 | No |
| 9 | PSRC1 |  |  | 574 | -0.295 | -0.3842 | No |
| 10 | DSCC1 |  |  | 596 | -0.299 | -0.3929 | No |
| 11 | RFC2 |  |  | 607 | -0.302 | -0.3927 | No |
| 12 | TMPO |  |  | 688 | -0.317 | -0.4479 | No |
| 13 | UBR7 |  |  | 767 | -0.336 | -0.5011 | No |
| 14 | MCM4 |  |  | 783 | -0.339 | -0.5038 | No |
| 15 | CDC25C |  |  | 792 | -0.341 | -0.5010 | No |
| 16 | CDC20 |  |  | 793 | -0.341 | -0.4917 | No |
| 17 | NCAPD2 |  |  | 811 | -0.347 | -0.4959 | No |
| 18 | BLM |  |  | 812 | -0.348 | -0.4864 | No |
| 19 | CBX5 |  |  | 842 | -0.356 | -0.4999 | No |
| 20 | GAS2L3 |  |  | 856 | -0.359 | -0.5006 | No |
| 21 | NEK2 |  |  | 861 | -0.359 | -0.4940 | No |
| 22 | CKAP2 |  |  | 900 | -0.375 | -0.5142 | No |
| 23 | TUBB4B |  |  | 916 | -0.380 | -0.5159 | No |
| 24 | MSH2 |  |  | 927 | -0.385 | -0.5134 | No |
| 25 | USP1 |  |  | 930 | -0.386 | -0.5045 | No |
| 26 | DTL |  |  | 956 | -0.397 | -0.5137 | No |
| 27 | CDC45 |  |  | 1000 | -0.413 | -0.5368 | No |
| 28 | POLD3 |  |  | 1002 | -0.415 | -0.5264 | No |
| 29 | KIF11 |  |  | 1003 | -0.416 | -0.5151 | No |
| 30 | CDC6 |  |  | 1008 | -0.418 | -0.5069 | No |
| 31 | TPX2 |  |  | 1034 | -0.437 | -0.5151 | No |
| 32 | BRIP1 |  |  | 1052 | -0.447 | -0.5165 | No |
| 33 | POLA1 |  |  | 1088 | -0.464 | -0.5319 | No |
| 34 | E2F8 |  |  | 1116 | -0.485 | -0.5403 | Yes |
| 35 | TTK |  |  | 1119 | -0.487 | -0.5287 | Yes |
| 36 | PCNA |  |  | 1142 | -0.504 | -0.5326 | Yes |
| 37 | CKAP2L |  |  | 1147 | -0.508 | -0.5220 | Yes |
| 38 | NUSAP1 |  |  | 1154 | -0.515 | -0.5128 | Yes |
| 39 | GMNN |  |  | 1155 | -0.516 | -0.4988 | Yes |
| 40 | RRM1 |  |  | 1166 | -0.534 | -0.4923 | Yes |
| 41 | KIF2C |  |  | 1183 | -0.558 | -0.4899 | Yes |
| 42 | CDCA3 |  |  | 1187 | -0.563 | -0.4771 | Yes |
| 43 | FEN1 |  |  | 1191 | -0.569 | -0.4640 | Yes |
| 44 | NUF2 |  |  | 1194 | -0.574 | -0.4501 | Yes |
| 45 | CKS1B |  |  | 1198 | -0.577 | -0.4368 | Yes |
| 46 | GTSE1 |  |  | 1210 | -0.602 | -0.4293 | Yes |
| 47 | CLSPN |  |  | 1212 | -0.605 | -0.4137 | Yes |
| 48 | SMC4 |  |  | 1224 | -0.638 | -0.4051 | Yes |
| 49 | CKS2 |  |  | 1226 | -0.641 | -0.3885 | Yes |
| 50 | ANLN |  |  | 1229 | -0.646 | -0.3726 | Yes |
| 51 | CDCA8 |  |  | 1239 | -0.669 | -0.3617 | Yes |
| 52 | CENPA |  |  | 1243 | -0.679 | -0.3456 | Yes |
| 53 | AURKB |  |  | 1250 | -0.700 | -0.3314 | Yes |
| 54 | CDCA2 |  |  | 1258 | -0.727 | -0.3173 | Yes |
| 55 | HMGB2 |  |  | 1263 | -0.736 | -0.3005 | Yes |
| 56 | RAD51AP1 |  |  | 1264 | -0.741 | -0.2805 | Yes |
| 57 | KIF23 |  |  | 1265 | -0.741 | -0.2604 | Yes |
| 58 | TACC3 |  |  | 1268 | -0.751 | -0.2416 | Yes |
| 59 | AURKA |  |  | 1271 | -0.763 | -0.2225 | Yes |
| 60 | TYMS |  |  | 1272 | -0.770 | -0.2016 | Yes |
| 61 | CCNE2 |  |  | 1273 | -0.771 | -0.1807 | Yes |
| 62 | ATAD2 |  |  | 1275 | -0.775 | -0.1605 | Yes |
| 63 | NDC80 |  |  | 1278 | -0.810 | -0.1401 | Yes |
| 64 | HJURP |  |  | 1290 | -0.897 | -0.1246 | Yes |
| 65 | RRM2 |  |  | 1295 | -0.933 | -0.1025 | Yes |
| 66 | CDK1 |  |  | 1311 | -1.433 | -0.0756 | Yes |
| 67 | UBE2C |  |  | 1312 | -1.466 | -0.0358 | Yes |
| 68 | TOP2A |  |  | 1313 | -1.528 | 0.0056 | Yes |
Table: GSEA details [plain text format]

  

Fig 2: SEURAT\_CELLCYCLE: Random ES distribution      
 Gene set null distribution of ES for **SEURAT\_CELLCYCLE**
